# Fragmented Dermo-Epidermal Units (FdeU) as an Emerging Strategy to Improve Wound Healing Process: An In Vitro Evaluation and a Pilot Clinical Study

**DOI:** 10.3390/jcm12196165

**Published:** 2023-09-24

**Authors:** Michele Riccio, Elena Bondioli, Letizia Senesi, Nicola Zingaretti, Paolo Gargiulo, Francesco De Francesco, Pier Camillo Parodi, Barbara Zavan

**Affiliations:** 1 Department of Reconstructive Surgery and Hand Surgery, University Hospital (AOU Ospedali Riuniti di Ancona), Via Conca 71, Torrette di Ancona, 60123 Ancona, Italy; michele.riccio@ospedaliriuniti.marche.it (M.R.); letizia.senesi@ospedaliriuniti.marche.it (L.S.); francesco.defrancesco@ospedaliriuniti.marche.it (F.D.F.); 2 Burn Center and Emilia Romagna Regional Skin Bank, Bufalini Hospital, AUSL della Romagna, 47521 Cesena, Italy; elena.bondioli@auslromagna.it; 3 Clinic of Plastic and Reconstructive Surgery, Academic Hospital of Udine, Department of Medical Area (DAME), University of Udine, 33100 Udine, Italy; zingarettin@gmail.com (N.Z.); piercamillo.parodi@uniud.it (P.C.P.); 4 Engineering Department, King’s College, London WC2R 2LS, UK; paolo@ru.is; 5 Institute for Biomedical and Neural Engineering, Reykjavík University, 101 Reykjavík, Iceland; 6 Department of Translational Medicine, University of Ferrara, Via Fossato di Mortara 70, 44121 Ferrara, Italy

**Keywords:** chronic wounds, micro-fragmented, Hy-Tissue, micrograft, regenerative dermal units

## Abstract

Innovative strategies have shown beneficial effects in healing wound management involving, however, a time-consuming and arduous process in clinical contexts. Micro-fragmented skin tissue acts as a slow-released natural scaffold and continuously delivers growth factors, and much other modulatory information, into the microenvironment surrounding damaged wounds by a paracrine function on the resident cells which supports the regenerative process. In this study, in vitro and in vivo investigations were conducted to ascertain improved effectiveness and velocity of the wound healing process with the application of fragmented dermo-epidermal units (FdeU), acquired via a novel medical device (Hy-Tissue^®^ Micrograft Technology). MTT test; LDH test; ELISA for growth factor investigation (IL) IL-2, IL-6, IL-7 IL-8, IL-10; IGF-1; adiponectin; Fibroblast Growth Factor (FGF); Vascular Endothelial Growth Factor (VEGF); and Tumor Necrosis Factor (TNF) were assessed. Therefore, clinical evaluation in 11 patients affected by Chronic Wounds (CW) and treated with FdeU were investigated. Functional outcome was assessed pre-operatory, 2 months after treatment (T0), and 6 months after treatment (T1) using the Wound Bed Score (WBS) and Vancouver Scar Scale (VSS). In this current study, we demonstrate the potential of resident cells to proliferate from the clusters of FdeU seeded in a monolayer that efficiently propagate the chronic wound. Furthermore, in this study we report how the discharge of trophic/reparative proteins are able to mediate the in vitro paracrine function of proliferation, migration, and contraction rate in fibroblasts and keratinocytes. Our investigations recommend FdeU as a favorable tool in wound healing, displaying in vitro growth-promoting potential to enhance current therapeutic mechanisms.

## 1. Introduction

Wound healing (WH) in adult mammals indicates the formation of a scar devoid of skin appendages. Scar formation is indeed able to impede infection and dehydration as a normal process but may meet with complications [1]. One of the most common complications in WH are Chronic Wounds (CW), defined as wounds that do not succeed to evolve through the normal phases of WH in an orderly and timely manner, where the process fails to restore functional integrity after three months. CW represent an economic burden for the healthcare system and a great challenge in terms of management and time [2]. Among CW, nonhealing ulcers, especially venous ulcers in the lower extremity, occur very frequently and affect the quality of life of patients, causing a social issue due to reduced mobility and social isolation [3]. In CW, the normal mechanism of healing is overturned [4]. They usually remain static in the inflammatory phase, with excessive secretion of proinflammatory cytokines, proteases, senescent cells, reactive oxygen species (ROS), persistent infection, and deficiency of stem cell production [5]. The aforementioned process is crucial to close the wound efficiently, but may adversely provoke fibrosis and skin scarring in CW. Most certainly, many peptide growth factors are crucial to correct wound healing [6,7]. Recently, regenerative medicine provided a fundamental breakthrough regarding CW and numerous pathologies, which lead to substance or volume loss or damage to tissues or organs [8,9,10]. Innovative strategies for promoting quality wound healing gained increasing interest in the scientific community because of their ability to accelerate wound healing, but in large burns, injury, or extensive chronic loss of substance, skin grafting is usually considered the principal procedure to sustain CWH as it is the cheapest and faster procedure.

However, adverse events related to skin grafting consist in graft dehiscence, partial graft loss, donor site morbidity, poor color matching, pain, discomfort, and hypertrophic scars [11,12]. To resolve this problem, Meek introduced a micrografting technique, which implied the division of the skin into small areas with a possibility of greater (ninefold) skin expansion [13]. In the original technique, pre-folded gauze was used to obtain a regular distribution of the autograft island via a Meek-Wall dermatome. However, the requirement of dermal orientation was unfeasible with such a small graft and the technique was eclipsed by mesh skin grafting, described by Tanner [14].

Kreis modified Meek’s original technique by using a dermatome turned on compressed air to obtain widely expanded postage stamp autografts [15]; the authors reported a great re-epithelization rate in 5 weeks (90%).

Numerous investigations have demonstrated [16,17,18,19] that micrografting is adequate for major burns (>30% TBSA) and in such cases where limited donor sites are unable to supply the necessary quantity of skin graft [20]. Novel procedures have been initiated due to biotechnological advances to gain autologous cellular suspensions during surgical interventions and to consent one-step management regarding acute and chronic skin lesions to enhance wound healing and regenerate normal skin architecture [21,22,23,24].

For example, ‘Rigenera micrograft technology’ can mechanically disaggregate autologous tissue, collecting autologous micrografts enriched in progenitor cells, growth factors, and particles of extracellular matrix derived from the patient’s own tissue [3]. In the literature, several independent papers described the employment of this technique for CWH [25,26,27,28], where 2 mL of final solution was put in a collagen scaffold in contact with the wound [28].

The aim of this paper is to demonstrate that fragmented dermo-epidermal units (FdeU), mechanically obtained from skin and one-step-processed by HY-TISSUE MICROGRAFT TECHNOLOGY, promote the repair of CW with key elements including natural scaffold (extracellular matrix structure), cells (fibroblasts), and growth factors (secreted cytokines and chemokines) to support the regenerative process, and thus represent a valid tool in chronic wound healing management, without blemishes and with minimal patient discomfort.

## 2. Materials and Methods

**Figure 1:**
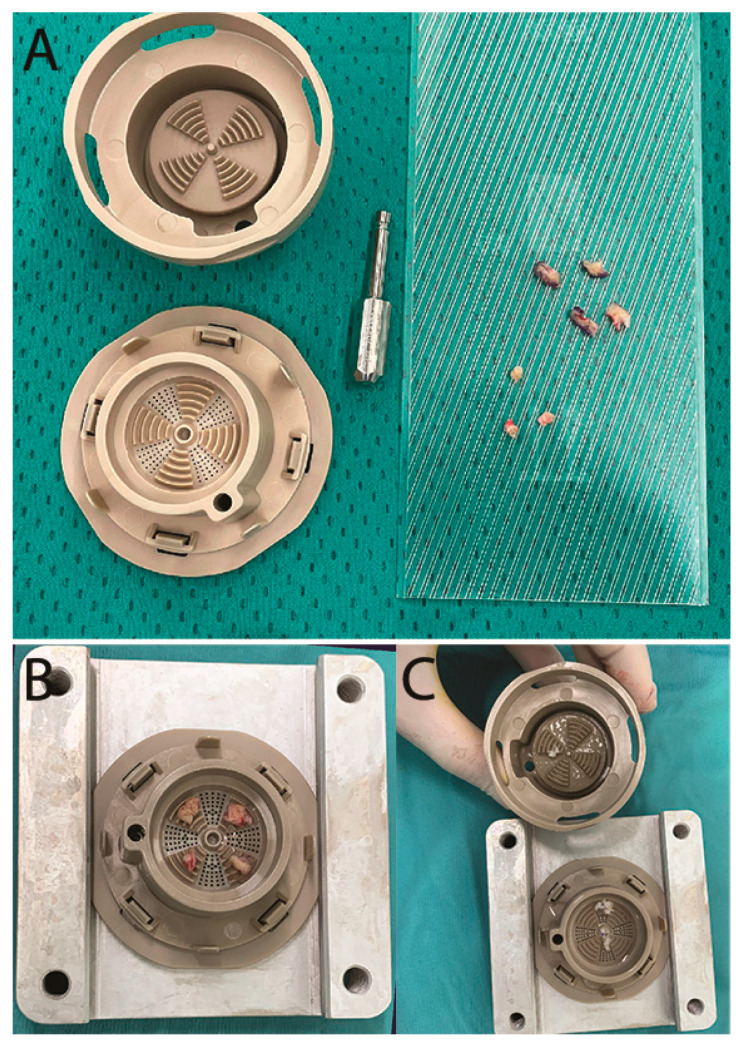
(**A**). Full-thickness skin-punch-biopsies of 5–6 mm avoid subdermal fat tissue; (**B**). placement of skin biopsies on the grid area of the device, which contained the fragmentation area (one by each quadrant); (**C**). the fragmented skin biopsies at the end of the procedure.

FdeU were obtained by means of HY-TISSUE MICROGRAFT TECHNOLOGY, a polyether-ether-ketone-made, single-use medical device for the fragmentation of tissue biopsies. The medical device is sterile and single use, and the technology was used in the operating room in sterile conditions. Full-thickness skin-punch-biopsies of 5–6 mm (devoid of subdermal fat tissue) were placed on the grid area of the device, which contained the fragmentation area (one by each quadrant—Figure 1). Saline solution (15 mL) was injected into the lower chamber to encounter the biopsies. Then, the upper portion of the device, equipped with a rotating cutting surface, was fixed on the lower base. Once the device was assembled, fragmentation occurred by rotation of the upper part of the device using a micro motor (rotation speed set at 150 rpm). Once a 400 µm dimension of the tissue fragment was obtained, it was passed through the grid and collected in the lower chamber. At the end of the process (up to 2 min), micrografts were collected from the lower chamber by aspiration with a 20 mL syringe without needle. 

### 2.1. In Vitro Study

For each patient (*n* = 11), cells obtained after treatment were evaluated, in triplicate, as follows:

#### 2.1.1. MTT Test Assay

Each skin biopsy (6 mm Ø) was divided into two portions. The first portion was dissected using the device according to manufacturing instructions. Following micro-fragmentation, the collected micrografts were concentrated by centrifugation and the pellet obtained was immersed in MTT solution. The second portion of skin biopsy was digested enzymatically in dispase solution (25 UI/mL) for 90 min at 37 °C. The enzymatic action was neutralized by adding complete medium. Gently separate the dermis from the epidermis with two pairs of forceps. Cut the dermis into very small pieces and transfer the pieces into a centrifugation tube containing 5 mL of collagenase A (Roche, Monza, MB, Italy) plus antibiotic/antimycotic for 4–8 h at 37 °C. The enzymatic action was neutralized by adding complete medium. Then, the sample was centrifuged at 200 rpm for 5 min, the supernatant was discarded, and resuspended in 1 mL of complete medium. The obtained cells were plated on a 25 cm^2^ T-flask and incubated at 37 °C with 5% CO_2_. Three days after the cells’ extraction, the complete medium was changed and then every 48 h until 80% confluence and used for the cell viability evaluation by MTT solution.

Uniform samples of skin obtained by a punch biopsy (6 mm Ø) were dissected using the device according to manufacturing instructions. To determine the presence of viable cells, the MTT assay was performed according to the method of Denizot and Lang with minor modifications. Briefly, the samples after treatment were seeded into 96 multiwell plates. After 21 days of culture, samples were incubated for 3 h at 37 °C in 50 μL of MTT solution. After the removal of the MTT solution, 150 μL of 10% DMSO in isopropanol was added for 30 min at 37 °C. 

The absorbance was read at 570 nm using a multilabel plate reader (Victor 3, Perkin Elmer, Milano, Italy).

Following micro-fragmentation, the collected micrografts were concentrated by centrifugation and the pellet obtained was immersed in MTT solution. Then, MTT was removed by centrifugation and the pelleted micrografts were re-suspended in DMSO solution. Absorbance at 570 nm was calculated to quantify viable cells. Similar non-dissected skin-punch biopsies were used as controls. To identify the optimal processing time, the maximum amount of skin tissue to be processed, the product reusability, and process reproducibility, punch biopsies were processed for 60, 120, or 180 s using the same device.

#### 2.1.2. Morphology with Electron Microscopy: SEM Analysis

To perform the test, uniform samples of skin obtained by a punch biopsy (6 mm Ø) were dissected using the device according to manufacturing instructions. Following micro-fragmentation, the collected micrografts were concentrated by centrifugation and the pellet obtained was dry in the fume hood for 24 h, assembled on metal stubs and sputter-coated with gold palladium, then analyzed under high vacuum conditions with the secondary electron detector.

SEM analysis of skin micrografts was conducted at Centro di Analisi e Servizi Per la Certificazione (CEASC, University of Padova, Padova, Italy) (SEM) with a FESEM, QUANTA200, (FEI, Eindhoven, The Netherlands) instrument. The samples were set to dry in the fume hood for 24 h, assembled on metal stubs, and sputter-coated with gold palladium, then analyzed under high vacuum conditions with the secondary electron detector.

#### 2.1.3. LDH Activity Assay

To perform the test, uniform samples of skin obtained by a punch biopsy (6 mm Ø) were dissected using the device according to manufacturing instructions. Following micro-fragmentation, the collected micrografts were concentrated by centrifugation and the pellet obtained was immersed in LDH solution.

LDH activity was assessed with the LDH Activity Assay Kit (Sigma-Aldrich, St. Louis, MI, USA) according to the manufacturer’s instructions at 0, 1, 3, 6, 25 h of samples on DMEM at 37 °C. The culture medium was promptly collected to identify extracellular LDH activity and the intracellular LDH activity was measured following cell lysis using the assay buffer in the kit. Each sample was incubated with a set reaction mixture, and the final product was evaluated at 450 nm using Victor 3 plate reader. All conditions were tested in duplicate.

#### 2.1.4. Citokine Production with ELISA Test

To perform the test, uniform samples of skin obtained by a punch biopsy (6 mm Ø) were dissected using the device according to manufacturing instructions. Following micro-fragmentation, the collected micrografts were concentrated by centrifugation and the pellet obtained used for ELISA test.

The supernatants of the samples were collected, centrifuged at 400× *g* for 10 min at 4 °C, then stored frozen for analysis of Interleukin (IL) IL-2, IL-6, IL-7 IL-8, IL-10; IGF-1; adiponectin; Fibroblast Growth Factor (FGF); Vascular Endothelial Growth Factor (VEGF); and Tumor Necrosis Factor (TNF) production using commercial ELISA kits (Thermo Fisher Scientific, Waltham, MA, USA) in conformity with the manufacturer’s instructions. Optical density (OD) values at 405 nm or 450 nm were estimated, using a Victor 3 plate reader. 

### 2.2. In Vivo Study

#### 2.2.1. Patients

This is a pilot, exploratory, one-center clinical study that is limited in size with the scope to give insight into the actions and efficacy of FdeU. Overall, 11 patients—5 females and 6 males—with a mean age of 48.2 years (mean 25–70 years), affected by CW (not healed within 3 months by the use of conventional therapy): post-traumatic wounds (7) and metabolic/vascular wounds (5), were treated with the micrograft technology. All patients/participants signed a written consent form to take part in the study in compliance with the Declaration of Helsinki. The AOU “Ospedali Riuniti” of Ancona Hospital Review Ethic Committee authorized the study (386/2022). No patients suffered from bone, vessel, nerve, or tendon exposure. In two cases, a micro-fragmented skin procedure was administered after treating the chronic wounds with a dermal regeneration template. Table 1 reports patient characteristics (with related pathologies). Wounds were regularly monitored at weekly intervals, while at an average 6-month follow-up, the evaluation of wound closure was completed.

#### 2.2.2. Surgical Procedure

**Figure 2:**
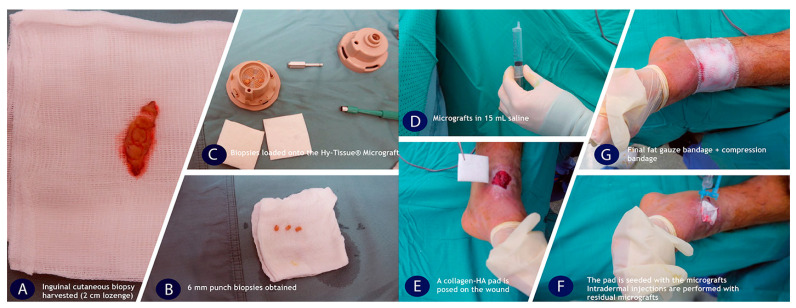
The figure showed surgical protocol of Hy-Tissue Micrograft: (**A**) Removal of a lozenge of skin tissue (from the groin or from the armpit); (**B**) Skin biopsy using a 6 mm punch biopsy instrument; (**C**) Placement of the skin biopsies onto the fragmentation area of the lower part; (**D**) After fragmentation, the micro-fragmented tissue is suspended in 15 mL saline solution; (**E**) Injection of 50% suspensions on a biological scaffold to create a regenerative bio-construct and set on the wound; (**F**) Injection of 50% suspension directly into the site of injury perilesionally; (**G**) Secondary moist dressing with paraffin gauze and moist gauze tie over.

All cases reported underwent treatment with FdeUs obtained by Hy-Tissue^®^ Micrograft (Fidia Farmaceutici, Abano Terme, Padova, Italy) after surgical or enzymatic ulcer debridement and following wound infection resolution probed by swab exam. Hy-Tissue Micrograft is a medical device able to produce a suspension of cutaneous autologous units (FdeU) that can be easily injected into the lesion alone or seeded combined with biological scaffolds (Bionect Pad or Hyalo4 Regen, Fidia Farmaceutici, Abano Terme, Padova, Italy) to improve the wound healing process. The technology is based on a mechanical disruption device able to reduce the skin into micro-fragments maintaining a constant size, with viable cells, thus allowing for expansion to cover the treated area. According to the protocol, biopsies were collected from either the inguinal or the axillary fold (in regions where the scar is hardly visible) under local anesthesia. After treatment with FdeUs, no patients received antibiotic therapy as the lesions had been adequately debrided, and the patients underwent three negative culture swabs before treatment. Only one patient received antibiotic therapy due to persistent positivity for Staphylococcus aureus. The first dressing change was performed after 6 days by removing the secondary dressing only. Wounds were subsequently assessed weekly to monitor healing progression. After complete healing, moisturizing oil was gently applied to the treated site and after one month the patient began tissue massage. The surgical protocol was defined following the different steps as reported in Figure 2:

(1) The adequate number of 6 mm punch biopsies were harvested (Kai Medical, Solingen, Germany) from the skin (devoid of subdermal fat tissue); the number of dermal punch biopsies was calculated according to the size of the wound, considering that 1 mm^2^ of the dermal micrografts was expected to heal a wound of maximum size 2 cm^2^;
(2) Punch skin biopsies were placed on the fragmentation area of the grid. No more than four pieces were processed simultaneously, one for each quadrant of the grid;
(3) Tissue processing was conducted (with a rotation speed of 150 rpm) through the addition of 15 mL of sterile physiological solution;
(4) Saline solution containing the micrografts was aspirated from the lower chamber when fragmentation was complete, using a 20 mL sterile syringe without the needle;
(5) Seeding of approximately 50% of suspensions into the scaffold (Bionect Pad or Hyalo4 Regen, Fidia Farmaceutici, Abano Terme, Padova, Italy) to create the bio-construct. Approximately 50% of suspension by 2 punch of 6 mm, resuspended in 15 mL of saline, can be seeded on a 5 × 5 cm^2^ dressing;
(6) Injection of the remaining suspensions into the lesion site of injury and placement of the bio-constructs over the ulcer;
(7) Secondary moist dressing with paraffin gauze and moist gauze on top with final moderate compressive dressing.

#### 2.2.3. Clinical Assessments

For each patient, the surface of the wounds at day 7 and then every week up to complete healing were measured and each wound was evaluated for contraction. Surfaces were monitored by tracing the wound edges using the acetate method with application of a two-layer transparent acetate over the wound and tracing of its perimeter. The contact layer of acetate was then set aside and the top layer, pre-printed with 1 cm^2^ squares, was utilized to measure the wound area. During the follow-up examinations, adverse events or complications were observed and recorded. Functional outcome was assessed pre-operatory, 2 months after treatment (T0), and 6 months after treatment (T1) using the Wound Bed score (WBS) (healing time, eschar, granulation tissue, exudate, dermatitis, fibrosis, wound bed) to determine prognosis and changes in management of wounds [29] during the treatment. Additionally, aesthetic appearance of the scar was evaluated using the Vancouver Scar Scale (VSS) (pigmentation, pliability, thickness, scar quality) after re-epithelialization [30,31].

### 2.3. Statistical Analysis

All experiments were performed in triplicates and the comparison was performed as disaggregated biopsies with FdeU vs. non disaggregated biopsies (control biopsy).

The Shapiro–Wilk test revealed abnormal distribution of data and consequently all statistical analyses were performed using a non-parametric approach. All data were statistically measured using the one-way ANOVA test. The threshold for statistical significance was set at *p*-values < 0.05.

## 3. Results

### 3.1. Definition of Parameters to Obtain FdeU

To evaluate the viability of the cells within the FdeU, MTT assay was performed at different treatment times. 

**Figure 3:**
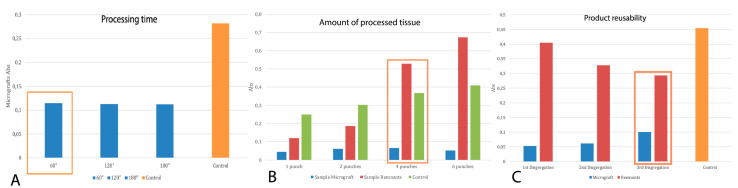
Protocol assessment by MTT test. (**A**) Overall, 60 s is sufficient to divide four biopsy punches and obtain the greatest number of viable cells; (**B**) The maximum number of biopsies that can be disrupted at the same time is 4; (**C**) The disruptor device can be reused three times while maintaining process reproducibility. Orange box shows the best result obtained.

As reported in Figure 3A, the higher cell viability was obtained after 60 s. The maximum amount of skin tissue that may dissected at 60 s was determined using MTT assay. Results, reported in Figure 3B, confirm that four punch-biopsies can be processed at once. The device can be reused three times while maintaining process reproducibility, as reported in Figure 3C.

### 3.2. Viability with MTT

**Figure 4:**
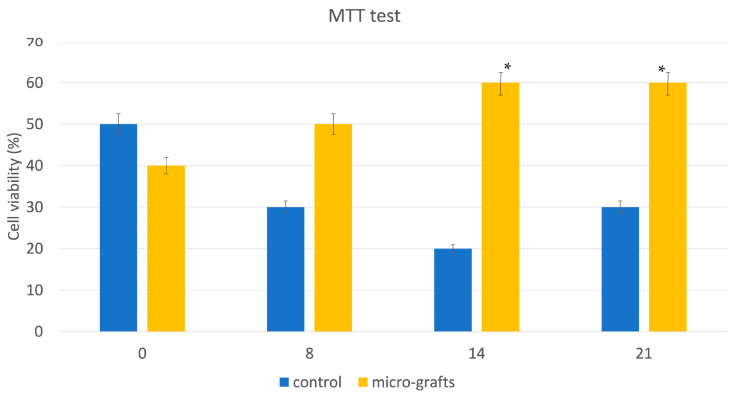
Viability assessment by MTT test showed that the fibroblasts obtained by enzymatic digestion (blue columns) show a decline in vitality (or metabolic activity) already after seven days of culture, unlike those obtained by mechanical disintegration (orange columns) which show a slow increase. * *p* < 0.005.

This system can maintain appreciable levels of cell viability after immediate disaggregation. The effect of disaggregation on cell viability was also investigated on tissue samples cultured for 8 days, 14 days, and 21 days. The results showed that a progressive variation (slow increase) of cell viability in micrograft samples was observed independently by time of homogenization compared to starting time (T0) (Figure 4). On the other hand, a reduced viability of cells obtained with enzyme digestion after 8 days of culture compared to starting time (T0) was observed, but the cell viability was restored after 21 days of culture (Figure 4).

### 3.3. Morphological Evaluation of FdeU

**Figure 5:**
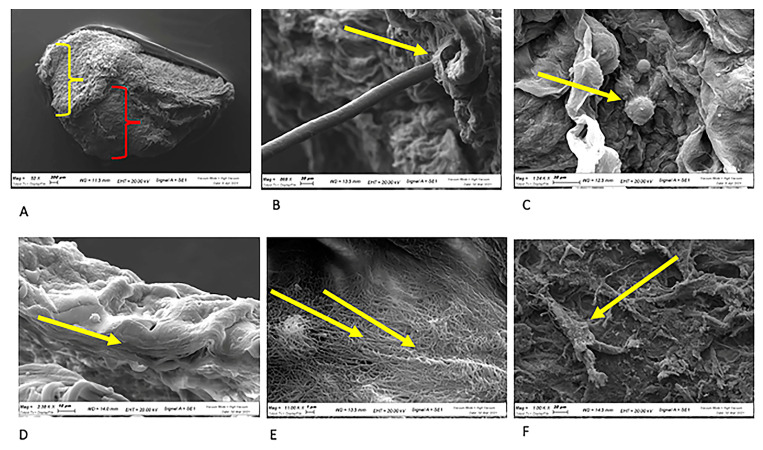
SEM characterization of FdeU. (**A**) Dermis where the superficial stratum corneum indicated by the yellow bracketed parenthesis is over a structured dermis (red bracketed parenthesis); (**B**) The horny layer containing the adnexa represented by the hairs, (yellow arrow); (**C**) Yellow arrow indicates round shape cells; (**D**) The cutaneous organization (Yellow arrow); (**E**) Collagen fibers (yellow arrows); (**F**) Fibroblasts (yellow arrow).

Morphological characterization of treated samples was conducted by electron microscopy. The specimen (Figure 5A) following treatment maintained the original structural organization typical of the dermis: superficial stratum corneum (yellow bracketed parenthesis) over a structured dermis (red bracketed parenthesis). The horny opposing layer also maintains the adnexa intact (hairs, Figure 5B yellow arrow), and is considered fundamental for tissue regeneration since the base contains the epidermal niche of the hair follicle, particularly rich in basal keratinocytes. Few round shape cells can be observed in the specimen (Figure 5C yellow arrow). Morphologically, the cutaneous organization with overlapping sheets is clearly preserved by the treatment (Figure 5D), while underneath, intertwined collagen fibers that are typical of the dermis (Figure 5E yellow arrows) and fibroblasts like cells. (Figure 5F yellow arrow) are observable.

### 3.4. Biological Evaluation of FdeU

**Figure 6:**
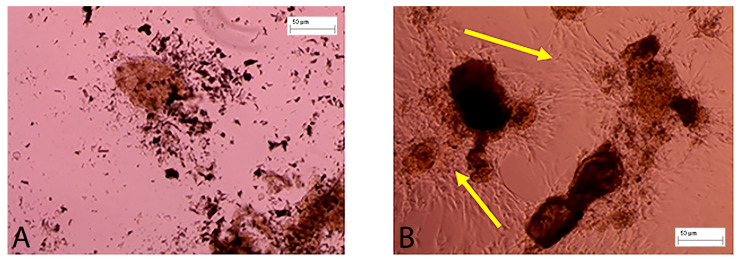
Cells present within the FdeU are able to spread and to colonize the plastic ware. (**A**) The figure shows micrografts seeded on plastic ware at time 0; (**B**) At day 8, cells with fibroblastoid morphology (yellow arrows) emerged from micrografts.

The presence of viable cells, potentially recruitable for in vivo healing promotion, was observed following in vitro seeding of the fragments obtained (Figure 6A). At culture day 8, cells with fibroblastoid morphology emerged from tissue fragments adhering to the culture plastic and able to proliferate (Figure 6B). This result confirms the potential of micrografts to proliferate and to allow the growth of fibroblastoid cells which are then responsible for the “colonization islands” present in the in vivo results. This concept is very important because wound healing usually occurs from the periphery (from the edges) and the use of micrografts also allows centrifugal healing (from the center).

**Figure 7:**
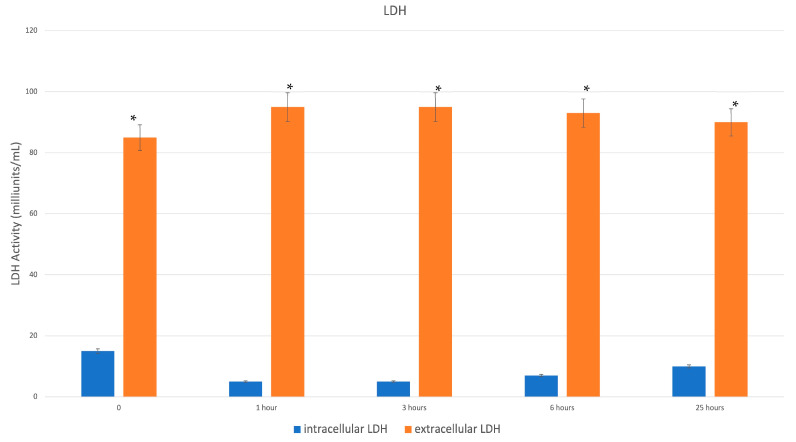
LDH test performed to evaluate cell membrane damage. The presence of LDH in the extracellular environment (orange columns) indicates damage at the cell membrane level. Blue columns indicate the intracellular LDH levels * *p* < 0.05.

**Figure 8:**
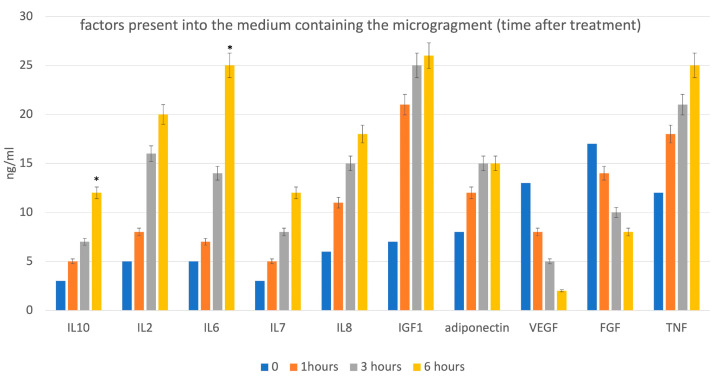
ELISA test performed on the medium containing the FdeU reveals the presence of growth factors. The levels of pro- and anti-inflammatory interleukins as well as growth factors secreted by cultured micrografts vary over time. * *p* < 0.05.

The presence of cells in tissues did not correspond to the total presence of viable cells. According to LDH assay, the treatment indeed induced damage to cell membranes, as shown in Figure 7. In fact, the intact cell confined the enzyme within the cytoplasm but membrane disruption (i.e., after tissue micro-fragmentation using the study device) regarding LDH enzyme activity was found in the micro-fragment supernatant. Biological activity of the FdeUs was evaluated by means of ELISA test for principal growth factors and in relation to interleukins. Results, reported in Figure 8, confirmed the presence of factors such as VEGF FGF and IGF-1. However, anti-inflammatory interleukins such as I-10, IL-7, and inflammatory factors such as IL-2 IL-6, IL-8, and TNF were also detected.

### 3.5. Clinical Results

**Figure 9:**
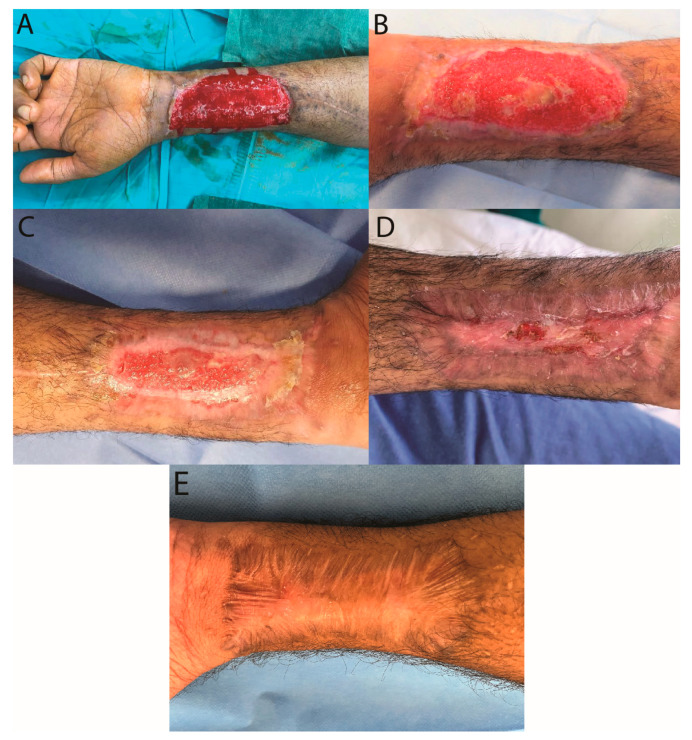
Clinical case of iatrogenic wound (free radial flap pick-up area). (**A**) Pre-operative view; (**B**) Follow-up visit 15 days after treatment: Central skin colonization island clearly visible, presumably developed following micrografts; (**C**) Image of the initial skin regeneration at follow-up examination 30 days after treatment; (**D**) Image of the complete skin regeneration at follow-up examination 45 days after treatment; (**E**) Image of complete skin regeneration at follow-up examination 6 months after treatment.

In this current study, representative cases among eleven patients admitted to our Plastic and Reconstructive Division were treated with autologous fragmented units obtained from processed skin. Follow-up of all patients was completed at 6 months. The mean age was 48.2 years (mean 25–70 years), with nine men (82%) and two women (18%). Seven (64%) patients presented ulcers located on the lower limb and four (36%) patients presented ulcers situated on the upper limb. Descriptions of patients and wounds are listed in Table 1. The origin of the soft tissue defect was a post-traumatic in six (55%) patients and metabolic/vascular wounds in five (45%) patients. In two patients, Hy-Tissue micrograft technology was used in combination with dermal substitute template (Integra^®^, Integra lifescience, Milan, Italy). Figure 9 shows a post-traumatic chronic ulcer of 45 cm^2^ (Figure 9A) treated with fragmented units seeded on a collagen-based dressing. An increase in the amount of granulation tissue was observed at the center of the wound with a peripheral fibrin layer after 15 days of follow-up (Figure 9B). After one month, a distinct reduction in wound size was noted, with new tissue and a significant contraction of the scar (Figure 9C) that was evident until the point of complete wound healing after 2 months (Figure 9D).

**Figure 10:**
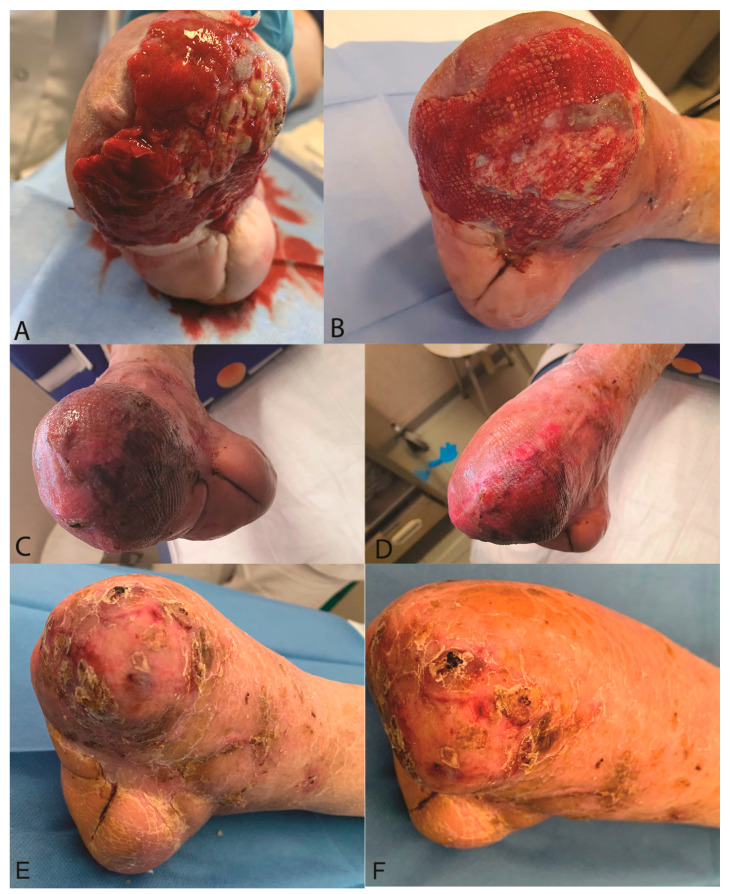
Clinical case of foot diabetic wound. (**A**) Pre-operative view; (**B**) Image of skin regeneration at follow-up examination 15 days after treatment; (**C**,**D**) Image of complete skin regeneration at follow-up examination 60 days after treatment; (**E**,**F**) Image of complete skin regeneration at follow-up examination 6 months after treatment.

Similar results were observed for a case such as diabetic ulcer (Figure 10). In all patients except one, an average complete recovery in 50 days was observed after the treatment with a range variable between 37 and 82 days. The treatment was unsuccessful in one patient, due to the presence of multidrug-resistant *Pseudomonas aeruginosa* infection, which required transfer to the infectious disease division of our hospital to initiate targeted antibiotic therapy. New wound debridement surgery was later required.

The VSS was adopted to assess functional and aesthetic characteristics of lesions at T0 and T1 follow-up as revealed in Table 2A. The VSS score defined a significant reduction at the T1 follow-up control examination (*p* < 0.05 *) for vascularity, pigmentation, and pliability. No statistical differences were noted for the height value (*p* = 0.60). The WBS score revealed a significant reduction at the T0 follow-up examination (*p* < 0.05 *) for healing edges, granulation tissue, oedema, and pink wound bed factors (Table 2B). At each follow-up visit, pain by visual analogue scale (VAS) was recorded to assess the amount of pain after the micrografts’ application. Patients reported a reduction in pain at each follow-up visit with a 5-point decrease from baseline (T0:6 and T1:1).

**Figure 11:**
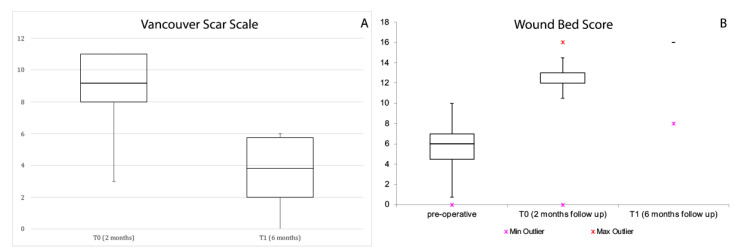
Variation of Vancouver (**A**) and WBS (**B**) total scores (squares, medians; bars, first and third quartiles). The reduction in the total score is significant (*p* < 0.05) in both cases.

Moreover, a remarkable reduction in all factors except black eschar was observed at the T1 follow-up examination (*p* = 0.00). The median VSS total score had decreased by 536 units (−58.4%) and the median WBS had increased by 58.9% (Figure 11). In addition, all patients expressed a relevant decrease in pain (data not reported) with no infection signs, allergic reactions, maceration, or inflammation around the lesion.

## 4. Discussion and Conclusions

CW typically present loss of substance and may be resistant to spontaneous healing, which entails vigilance regarding super-infection and exudation of the wounds [32,33]. Persistent progression of the lesion and later ulceration may be common complications. Such wounds are frequently treated with advanced dressings involving hyaluronic acid, hydrocolloids, proteolytic enzymes, hydrogels, and VAC therapy [34,35]. Several investigations have examined the effectiveness of regenerative medical techniques regarding next-generation biomaterials to regenerate an adequate healing environment [36,37,38,39]. Previous studies revealed that micro fragmented tissue used in post-traumatic vascular and metabolic CW is able to stimulate skin regeneration through a real regenerative process [25,26,27,28,40]. De Francesco et al. showed that dermal micrografts obtained from mechanical disruption improve the process of tissue repair in venous, diabetic, pressure, and post-traumatic ulcers, with a reduction in wound size, an increased granulation, and reduced exudation [27]. Riccio et al. demonstrated a significant diameter reduction in post-traumatic chronic leg ulcer treated with a micrograft [28]. In light of these findings, the aim of this current study is to evaluate the efficacy of a new device in producing micro-fragmented tissue, which is able to conserve biological properties. 

Comparing our study with those pre-existing in literature, we showed that cell viability and immunohistochemical expression are overlapping with Rigenera technology.

A definitive wound healing was achieved in 35–84 days with Rigenera technology [28] and in 37–82 days with Hy-tissue micrograft technology. Wound Bed Score was significantly lower after 12 months concerning healing hedges, exudates, and depth granulation tissue [28], while with Hy-Tissue technology it was significantly lower after 6 months concerning healing edge, granulation tissue, and pink wound bed. The mechanical devices seem to have similar results even if few studies are present in the literature to prove it.

Apart from that, two fundamental aspects emerged from the results of this study: 1. the maintenance of fibroblast metabolic activity, and 2. the maintenance of microscopic characteristics of micrografts obtained by Hy tissue technology. Maintaining fibroblast viability allows to obtain sterile autologous micrografts in a single step, avoiding extensive manipulations. Notably, in this current study, skin biopsies, treated with mechanical fragmentation showed an increase in cell viability after 8 days of culture, due to growth factors released into the culture medium. In addition to maintaining fibroblast viability, this system is safe and easy to use, and is capable of mechanically disaggregating skin tissue within minutes, preventing the breakdown of cellular structures compared to traditional methods of cell isolation, such as enzymatic digestion. Furthermore, this system preserves the extracellular matrix, as demonstrated by the ultrastructural images, which provides support to the gradual growth of the fibroblasts contained in the micrografts. On the contrary, the enzymatic digestion method induces an early drop in cellular metabolic activity/vitality and a later restoration of proliferation. It is noteworthy that the LDH assay result indicates a potential increase in cell death in FdeU samples since LDH release is considered a sign of cell membrane damage. This result seems to be in contrast with the high metabolic activity revealed by MTT assay. These apparently contradictory results can be interpreted in different ways: (1) the disruptor device damages a part of the cells causing LDH release, but contemporarily, the high MTT levels can be considered as the capability of the survived cells to counteract this cell loss; (2) cells obtained by mechanical disaggregation have an increase in anaerobic metabolism that causes the reported LDH increase. This happens when cells have an urgent need of ATP and glycolysis, representing a fast although inefficient way to product ATP. This metabolic perturbation in turn causes an oxygen debt in the cells that could be compensated by the increased activity of NAD(P)H-dependent cellular oxidoreductase enzymes (and this could even explain the contemporary increase in MTT assay positive signal). In any case, what happens is the trigger of a virtuous cycle of cell turnover that can be exploited for therapeutic purposes. It is fair to say that further investigations are needed to better understand this aspect.

It is important to note how the FdeU obtained from mechanical disaggregation maintain their histological ultrastructure made up of epidermis and structured dermis where two important components were preserved: adnexa (hair follicle) and the matrix made up of collagen fibers. These two elements are the basis of the so-called regenerative niche capable of the tissue regeneration property of micro-fragments. The in vitro results confirmed that the isolated tissue units, rich in collagen matrix useful for 3D proliferation of autologous cells, contain anti-inflammatory factors which may appropriately drive repair of damaged tissue and enhance growth factors required for capillary reorganization and synthesis of new matrix. A single cytokine may be secreted by different cells and can have either pro-inflammatory or anti-inflammatory actions depending on the framework, producing distinctive reactions. Consequently, a dynamic and ever-shifting balance between pro- and anti-inflammatory cytokines plays a substantial job in wound healing through mediating and regulating inflammation. Proinflammatory cytokines are the first factors to be produced in response to hard-to-heal wounds, and they control the roles of immune cells in epithelialization. Proinflammatory cytokines, mainly including tumor necrosis factor (TNF), interleukin (IL)-1, and IL-6, participate in the inflammation phase of wound healing through activating downstream cascades [41], and IL-7 can expand the local cells, enhancing proliferation and mobilization [42]; in fact, our studies reveal an increase in these proinflammatory factors in response to stress, in the initial phase. However, excessive production of proinflammatory cytokines is harmful and balance is a fundamental in wound healing. For these reasons, the above-mentioned proinflammatory cytokines are responsible for the phenotype transition from proinflammatory M1 macrophages to reparative M2 macrophages, and thus in the transition from the inflammatory phase to the proliferative phase [43]. M2 macrophages, which regulate inflammation, express anti-inflammatory mediators such as IL-10, as well as several growth factors to promote fibroblast proliferation, extracellular matrix synthesis, and angiogenesis. In particular, IL-10 has been demonstrated to be critical in healing without scarring [44] due to its action in ECM remodeling. In this phase, IL-10 is also responsible for the reduction in the expression of proinflammatory mediators [45]. 

This treatment results in granulation tissue formation (within 30 days), with tissue repair providing good wound healing with good aesthetic appearance, even in the presence of modest scar fibrosis. This current study revealed an increase in the cure rate in relation to the preoperative situation. Furthermore, a clinical evaluation of the skin trophism was conducted during the follow-up sessions, with significant improvements observed compared to the baseline properties. The results are in line with previous studies, revealing the structural remodeling potential of PRP [46], leading to the disposal of hyaluronic acid and glycosaminoglycan expression as well as modulate matrix metalloproteinase and tissue inhibitor metalloproteinase equilibrium [47,48]. 

Consequently, the case series herein described demonstrated a positive outlook following treatment with micro-fragmented factors regarding re-epithelization rate, prompt development of granulation tissue, and favorable recovery in blood supply. Moreover, the nature of micro-fragmented tissue as a complex matrix of extracellular components and soluble factors is noteworthy; it seems to promote a rapid generation of granulated tissue and supports shorter healing time and the necessity to recur to surgical interventions for reconstruction. Moreover, the outcomes regarding the healing process are associated with reduced hospitalization and healthcare expenses. Body repair mechanisms are thus stimulated to heal the damaged tissues. The main advantage of FdeUs is that wound closure may be enhanced or activated, committing a small quantity of donor tissue or at least less than what would be required in treatments involving split-thickness skin grafts. The concept of “foci of colonization” is therefore proposed, which represents the micrografts engaging with the wound bed and then slowly expanding through cell proliferation. The micrograft acted as a bio-stimulator, containing small quantities of fibroblastoid cells, annexa, and collagen fibers capable of stimulating the regenerative process at the site of the wound, thanks to the activity of secreted growth factors and pro- and anti-inflammatory cytokines. The Hy-Tissue micrograft was able to provide a positive microenvironment within the patient by offering specific signals, as shown above, capable of stimulating the regenerative process and therefore the healing of the chronic wound.

Nevertheless, our investigation presents limitations owing to the small sample size and lack of masking within the study, which may be considered a crucial point. Micro-fragments are fundamental in damaged tissue regeneration in CW, but several issues remain unclear, such as a better definition of the specific cell population of these micrografts, including the presence of progenitor cells or the role of the extracellular matrix and growth factors. Finally, a better clarification is needed to understand the metabolic activity of micrografts and how this activity is related to the bioreactor function for molecular processes that lead to healing of the wound. Future studies regarding the role of micro-fragmented skin in CW recovery are urgently required with an attention to specific perspectives as dermatology, vulnology, and trauma. 

In conclusion, our analysis concerning autologous micro-fragmented skin has yielded positive outcomes and confirms this may be considered a feasible therapeutic alternative to significantly ameliorate CW healing, as already reported in the literature.

## Figures and Tables

**Table 1:** Patient characteristics and wound features are shown.

	N (%)	Mean Value	Minimum–Maximum
Patients	11 (100%)		
Sex			
Male	9 (82%)		
Female	2 (18%)		
Age		48.2 years	25–70 years
Underlying disease			
None	4 (37%)		
Diabetes Mellitus type II	2 (18%)		
Hypertension	3 (27%)		
Arteriopathy	2 (18%)		
Wound location			
Upper Limb	4 (36%)		
Lower Limb	7 (64%)		
Type of injury			
Post-traumatic ulcer	6 (55%)		
Metabolic/vascular ulcer	5 (45%)		
Surface area		25 cm^2^	10–45 cm^2^
Exposed structures			
None	11 (100%)		
Tendon	0 (0%)		
Bone	0 (0%)		
Infection			
Before treatment	0 (0%)		
After treatment	1 (9%)		
Antibiotics			
Preoperative prophylaxis	11 (100%)		
Postop targeted AB	1 (9%)		
Time of complete healing		50 days	37–82 days
Complication/note			
None	8 (73%)		
Complicated infection	1 (9%)		
Associate dermal template	2 (18%)		

**Table 2:** (**A**). Vancouver Scar Scale (VSS) results. (**B**). Wound Bed Scale (WBS) results.

(A)					
	Pre-Treatment	T0 (2 Months Follow-Up)	T1 (6 Months Follow-Up)	*p*-Value	
Vascularity		2.01	0.54	*p = 0.00*	
Pigmentation		1.75	0	*p = 0.00*	
Pliability		3.72	2	*p = 0.0021*	
Height		1.45	1.27	*p* = 0.60	
Total Score		9.2	3.9		
**(B)**					
	**Pre-Treatment**	**T0** **(2 Months Follow-Up)**	***p*-Value**	**T1** **(6 Months Follow-Up)**	***p*-Value**
Healing edges	0.27	1.54	*p = 0.002*	1.81	*p = 0.00*
Black eschar	1.27	1.63	*p* = 0.332	1.81	*p* = 0.08
Depth/granulation tissue	0.45	1.45	*p = 0.0021*	1.81	*p = 0.00*
Exudate amount	1.09	1.63	*p* = 0.10	1.81	*p = 0.007*
Edema	0.54	1.54	*p = 0.0028*	1.81	*p = 0.00*
Periwound dermatitis	0.54	1	*p* = 0.08	1.81	*p = 0.00*
Periwound callus/fibrosis	0.54	1	*p* = 0.08	1.81	*p = 0.00*
Pink wound bed	0.54	1.18	*p = 0.031*	1.81	*p = 0.00*
Total score	5.27	11	*p = 0.007*	14.54	*p = 0.00*

## Data Availability

The datasets generated during and/or analysed during the current study are available from the corresponding author upon reasonable request.

## References

[1] Takeo M., Lee W., Ito M. (2015). Wound healing and skin regeneration. Cold Spring Harb. Perspect. Med.

[2] Olsson M., Järbrink K., Divakar U., Bajpai R., Upton Z., Schmidtchen A., Car J. (2019). The humanistic and economic burden of chronic wounds: A systematic review. Wound Repair Regen..

[3] Astarita C., Arora C.L., Trovato L. (2020). Tissue regeneration: An overview from stem cells to micrografts. J. Int. Med. Res..

[4] Martin P., Nunan R. (2015). Cellular and molecular mechanisms of repair in acute and chronic wound healing. Br. J. Dermatol..

[5] Levy V., Lindon C., Zheng Y., Harfe B.D., Morgan B.A. (2007). Epidermal stem cells arise from the hair follicle after wounding. FASEB J..

[6] Demidova-Rice T.N., Hamblin M.R., Herman I.M. (2012). Acute and impaired wound healing: Pathophysiology and current methods for drug delivery, part 2: Role of growth factors in normal and pathological wound healing: Therapeutic potential and methods of delivery. Adv. Skin Wound Care.

[7] Caley M.P., Martins V.L.C., O’Toole E.A. (2015). Metalloproteinases and wound healing. Adv. Wound Care.

[8] Klontzas M.E., Protonotarios A. (2021). High-resolution imaging for the analysis and reconstruction of 3D microenvironments for regenerative medicine: An application-focused review. Bioengineering.

[9] Saini G., Segaran N., Mayer J.L., Saini A., Albadawi H., Oklu R. (2021). Applications of 3D bioprinting in tissue engineering and regenerative medicine. J. Clin. Med..

[10] Lee J.Y., Kim H.S. (2021). Extracellular vesicles in regenerative medicine: Potentials and challenges. Tissue Eng. Regen. Med..

[11] You H.J., Han S.K. (2014). Cell therapy for wound healing. J. Korean Med. Sci..

[12] Darrach H., Kokosis G., Bridgham K., Stone J.P., Lange J.R., Sacks J.M. (2019). Comparison of keystone flaps and skin grafts for oncologic reconstruction: A retrospective review. J. Surg. Oncol..

[13] Meek C.P. (1958). Successful microdermagrafting using the Meek-Wall microdermatome. Am. J. Surg..

[14] Tanner J.C., Vandeput J., Olley J.F. (1964). The mesh skin graft. Plast. Reconstr. Surg..

[15] Kreis R.W., Mackie D.P., Vloemans A.W., Hermans R.P., Hoekstra M.J. (1993). Widely expanded postage stamp skin grafts using modified Meek technique in combination with an allograft overlay. Burns.

[16] Trovato L., Monti M., Del Fante C., Cervio M., Lampinen M., Ambrosio L., Redi C.A., Perotti C., Kankuri E., Ambrosio G. (2015). A New Medical Device Rigeneracons Allows to Obtain Viable Micro-Grafts From Mechanical Disaggregation of Human Tissues. J. Cell. Physiol..

[17] Meek C.P. (1963). Extensive severe burn treated with enzymatic debridement and microdermagrafting: Case report. Am. Surg..

[18] Hsieh C.S., Schuong J.Y., Huang W.S., Huang T.T. (2008). Five years’ experience of the modified Meek technique in the management of extensive burns. Burns.

[19] Quintero E.C., Machado J.F.W., Robles R.A.D. (2018). Meek micrografting history, indications, technique, physiology and experience: A review article. J. Wound Care.

[20] Dahmardehei M., Vaghardoost R., Saboury M., Zarei H., Saboury S., Molaei M., Seyyedi J., Maleknejad A. (2020). Comparison of Modified Meek Technique with Standard Mesh Method in Patients with Third Degree Burns. World J. Plast. Surg..

[21] Turner N.J., Badylak S.F. (2015). The use of biologic scaffolds in the treatment of chronic nonhealing wounds. Adv. Wound Care.

[22] James S.E., Booth S., Dheansa B., Mann D.J., Reid M.J., Shevchenko R.V., Gilbert P.M. (2010). Sprayed cultured autologous keratinocytes used alone or in combination with meshed autografts to accelerate wound closure in difficult-to-heal burns patients. Burns.

[23] Harkin D.G., Dawson R.A., Upton Z. (2006). Optimized delivery of skin keratinocytes by aerosolization and suspension in fibrin tissue adhesive. Wound Repair Regen..

[24] Jimi S., Takagi S., De Francesco F., Miyazaki M., Saparov A. (2020). Acceleration of Skin Wound-Healing Reactions by Autologous Micrograft Tissue Suspension. Medicina.

[25] De Francesco F., Graziano A., Trovato L., Ceccarelli G., Romano M., Marcarelli M., Cusella De Angelis G.M., Cillo U., Riccio M., Ferraro G.A. (2017). A Regenerative Approach with Dermal Micrografts in the Treatment of Chronic Ulcers. Stem Cell Rev. Rep..

[26] Trovato L., Failla G., Serantoni S., Palumbo F.P. (2016). Regenerative surgery in the management of the leg ulcers. J. Cell Sci. Ther..

[27] Miranda R., Farina E., Farina M.A. (2018). Micrografting chronic lower extremity ulcers with mechanically disaggregated skin using a micrograft preparation system. J. Wound Care.

[28] Riccio M., Marchesini A., Zingaretti N., Carella S., Senesi L., Onesti M.G., Parodi P.C., Ribuffo D., Vaienti L., De Francesco F. (2019). A Multicentre Study: The Use of Micrografts in the Reconstruction of Full-Thickness Posttraumatic Skin Defects of the Limbs-A Whole Innovative Concept in Regenerative Surgery. Stem Cells Int..

[29] Falanga V., Saap L.J., Ozonoff A. (2006). Wound Bed score and its correlation with healing of chronic wounds. Dermatol. Ther..

[30] Vercelli S., Ferriero G., Sartorio F., Stissi V., Franchignoni F. (2009). How to assess postsurgical scars: A review of outcomes measures. Disabil. Rehabil..

[31] Draaijers L.L., Tempelman F.R.H., Botman Y.A.M., Tuinebreijer W.E., Middelkoop E., Kreis R.W., van Thompson C.M., Sood R.F., Honari S., Carrougher G.J. (2015). What score on the Vancouver Scar Scale constitutes a hypertrophic scar? Results from a survey of North American burn-care providers. Burns.

[32] Frank C., Bayoumi I., Westendorp C. (2005). Approach to infected skin ulcers. Can. Fam. Physician.

[33] Vowden K., Vowden P. (2003). Understanding exudate management and the role of exudate in the healing process. Br. J. Community Nurs..

[34] Heyer K., Augustin M., Protz K., Herberger K., Spehr C., Rustenbach S.J. (2013). Effectiveness of advanced versus conventional wound dressings on healing of chronic wounds: Systematic review and meta-analysis. Dermatology.

[35] Powers J.G., Morton L.M., Phillips T.J. (2013). Dressings for chronic wounds. Dermatol. Ther..

[36] Rodrigues M., Kosaric N., Bonham C.A., Gurtner G.C. (2019). Wound healing: A cellular perspective. Physiol. Rev..

[37] Gurtner G.C., Chapman M.A. (2016). Regenerative medicine: Charting a new course in wound healing. Adv. Wound Care.

[38] Cottone G., Amendola F., Strada C., Bagnato M.C., Brambilla R., De Francesco F., Vaienti L. (2021). Comparison of Efficacy among three dermal substitutes in the management of critical lower-limb wounds: The largest biases-reduced single-center retrospective cohort study in literature. Medicina.

[39] De Francesco F., Busato A., Mannucci S., Zingaretti N., Cottone G., Amendola F., De Francesco M., Merigo F., Riccio V., Vaienti L. (2020). Artificial dermal substitutes for tissue regeneration: Comparison of the clinical outcomes and histological findings of two templates. J. Int. Med. Res..

[40] Marcarelli M., Trovato L., Novarese E., Riccio M., Graziano A. (2017). Rigenera protocol in the treatment of surgical wound dehiscence. Int. Wound J..

[41] Xiao T., Yan Z., Xiao S., Xia Y. (2020). Proinflammatory cytokines regulate epidermal cells in wound epithelialization. Stem Cells Res. Ther..

[42] Bartlett A., Sanders A.J., Ruge F., Harding K.G., Jiang W.G. (2016). Potential implications of interleukin-7 in chronic wound healing. Exp. Ther. Med..

[43] Landen N.X., Li D., Stahle M. (2016). Transition from inflammation to proliferation: A critical step during wound healing. Cell. Mol. Life Sci..

[44] Singampalli K.L., Balaji S., Wang X., Parikh U.M., Kaul A., Gilley J., Birla R.K., Bollyky P.L., Keswani S.G. (2020). The role of an IL-10/hyaluronan axis in dermal wound healing. Front. Cell Dev. Biol..

[45] Peranteau W.H., Zhang L., Muvarak N., Badillo A.T., Radu A., Zoltick P.W., Liechty K.W. (2008). IL-10 overexpression decreases inflammatory mediantors and promotes regenerative healing in an adult model of scar formation. J. Investig. Dermatol..

[46] Everts P., Onishi K., Jayaram P., Lana J.F., Mautner K. (2020). Platelet-Rich Plasma: New performance understandings and therapeutic considerations in 2020. Int. J. Mol. Sci..

[47] De Francesco F., Riccio M., Jimi S. (2022). Contribution of topical agents such as Hyaluronic acid and silver sulfadiazine to wound healing and management of bacterial biofilm. Medicina.

[48] De Francesco F., De Francesco M., Riccio M. (2022). Hyaluronic acid/collagenase ointment in the treatment of chronic hard-to-heal wounds: An observational and retrospective study. J. Clin. Med..

